# Topoisomerase 1 Activity Is Reduced in Response to Thermal Stress in Fruit Flies and in Human HeLa Cells

**DOI:** 10.3390/bios13110950

**Published:** 2023-10-24

**Authors:** Trine Juul-Kristensen, Josephine Geertsen Keller, Kathrine Nygaard Borg, Noriko Y. Hansen, Amalie Foldager, Rasmus Ladegaard, Yi-Ping Ho, Volker Loeschcke, Birgitta R. Knudsen

**Affiliations:** 1 Department of Molecular Biology and Genetics, Aarhus University, 8000 Aarhus, Denmark; tjk@mbg.au.dk (T.J.-K.); jgk@mbg.au.dk (J.G.K.); kborg@link.cuhk.edu.hk (K.N.B.); noriko@mbg.au.dk (N.Y.H.); afo@clin.au.dk (A.F.); ladegaardmadsen@gmail.com (R.L.); 2 Department of Biomedical Engineering, The Chinese University of Hong Kong, Hong Kong SAR, China; ypho@cuhk.edu.hk; 3 Centre for Biomaterials, The Chinese University of Hong Kong, Hong Kong SAR, China; 4 Hong Kong Branch of CAS Center for Excellence in Animal Evolution and Genetics, Hong Kong SAR, China; 5 State Key Laboratory of Marine Pollution, City University of Hong Kong, Hong Kong SAR, China; 6 Department of Biology, Aarhus University, 8000 Aarhus, Denmark

**Keywords:** topoisomerase 1, thermal stress, enzyme activity, biosensor, biomarker

## Abstract

In the modern world with climate changes and increasing pollution, different types of stress are becoming an increasing challenge. Hence, the identification of reliable biomarkers of stress and accessible sensors to measure such biomarkers are attracting increasing attention. In the current study, we demonstrate that the activity, but not the expression, of the ubiquitous enzyme topoisomerase 1 (TOP1), as measured in crude cell extracts by the REEAD sensor system, is markedly reduced in response to thermal stress in both fruit flies (*Drosophila melanogaster*) and cultivated human cells. This effect was observed in response to both mild-to-moderate long-term heat stress and more severe short-term heat stress in *D. melanogaster*. In cultivated HeLa cells a reduced TOP1 activity was observed in response to both cold and heat stress. The reduced TOP1 activity appeared dependent on one or more cellular pathways since the activity of purified TOP1 was unaffected by the utilized stress temperatures. We demonstrate successful quantitative measurement of TOP1 activity using an easily accessible chemiluminescence readout for REEAD pointing towards a sensor system suitable for point-of-care assessment of stress responses based on TOP1 as a biomarker.

## 1. Introduction

The potentially harmful effects of stress in response to various environmental influences have gained increasing interest during the past decades, where increasing temperatures, drought, or flood associated with the ongoing climate changes challenge livestock and crops [1,2,3,4]. With this, the identification of valid biomarkers and easily accessible sensor systems for monitoring stress is becoming more and more urgent [5,6,7,8,9].

At the cellular level, stress responses can be defined as the cellular reactions necessary for the rescue of essential metabolic processes after exposure to extrinsic or intrinsic stressors [10,11,12]. It comprises a mechanism that is evolutionarily highly conserved among species [13,14,15,16,17,18,19,20,21,22,23,24,25,26,27,28,29,30,31,32,33,34,35]. Indeed, the stress-induced reactions appear to be so interlinked that the generation of one type of stress response may influence another leading to overlapping or even identical cellular changes in response to very different stressors. Consequently, it may be possible to identify common markers for stress in general, or at least for a broad range of different stress conditions.

Until now, stress responses have primarily been addressed at the physiological level and it is relatively clear that different stressors induce physiological and endocrine alterations that may be evident in changes in the biochemical (e.g., hormones) or metabolic systems (affecting e.g., metabolites, enzymes or hormones) [36,37]. Moreover, the available evidence suggests that cellular stress responses are associated with alterations in protein expression or activity levels as well as in essential DNA processes that appear conserved between species [23,38,39,40,41]. The aim of our study is to start a comparison to test if one single biomarker can be used across species from human cell cultures to fruit flies on one side and across different stress types of varying lengths and intensities on the other side.

Several classes of potential biomarkers for a broad range of different types of stress have been identified [2,42,43,44]. Besides well-known chemical secretions such as cortisol and α-amylase, these include a large group of chaperone proteins, the heat shock proteins (hsp), that are induced in response to many different stressors, innate immune markers, the so-called acute phase proteins (APPs), and oxidative stress markers [23,45,46,47]. It is evident that similarities in the response to different stressors can be observed at the cellular level. However, most cellular markers identified so far vary with respect to half-life [11,48]. Also, most markers have been identified and validated in studies of acute or severe stress [10,11], and information on the cellular response towards long-term low to moderate stress exposure is still inadequate.

A study by Velichko et al. (2015) implicated the nuclear enzyme, topoisomerase I (TOP1) as a marker for acute sublethal heat stress in cultivated HeLa cells [49]. Eukaryotic TOP1 is a ubiquitous enzyme that is constitutively expressed and active throughout the cell cycle [50]. It regulates DNA topology by introducing transient single-strand breaks in the DNA backbone via the formation of a covalent TOP1-DNA cleavage complex (TOP1cc). In the duration of this intermediate TOP1cc, the cleaved strand can rotate around the un-cleaved strand to relieve torsional stress. Thereafter, the integrity of the DNA backbone is restored in a re-ligation step. Under normal circumstances, the catalytic equilibrium of TOP1 is shifted toward re-ligation to preserve the integrity of the genetic material. However, the equilibrium can be changed under certain circumstances for instance under the influence of the TOP1-specific anti-cancer agent camptothecin (CPT) and its derivatives. These drugs act by inhibiting the re-ligation reaction and thereby causing accumulation of TOP1cc [50,51]. Upon collision with the replication machinery, such TOP1cc is converted to permanent double-strand breaks (DSBs) resulting in senescence and cell death [51]. A reminiscent phenotype, involving TOP1cc-dependent generation of DSBs and subsequent senescence, was reported by Velichko et al. (2015) in cultured HeLa cells exposed to acute sublethal heat stress at 45.5 °C for 30 min [49]. This was accompanied by a dramatic reduction in TOP1 activity levels, which was also observed by Ciavarra et al. in previous studies [52,53]. However, neither of the studies addressed the effect of moderate long-term heat stress on TOP1 activity in cultured cells. Combined the data suggest that TOP1 may be a player in the cellular heat stress response and the induction of TOP1cc was addressed at various temperatures by Velichko et al. (2015) [49]. In this study the TOP1cc-induced DSBs and senescence were only observed upon acute sublethal heat stress and were not induced in cells exposed to mild (41 °C) or moderately (43 °C) increased temperatures for 30 min. The TOP1 activity levels, however, were never addressed in cells exposed to heat stress temperatures below 44 °C in any of the three studies [49,52,53].

In the current study we have measured TOP1 activity at the single catalytic event level using the previously described REEAD sensor system [54] in extracts from human cells and *D. melanogaster* subjected to various degrees of thermal stress, including mild long-term heat stress. This is the first time TOP1 activity is measured in an organism (*D. melanogaster*) subjected to short-term acute or long-term moderate heat stress and in a cell line (HeLa) in response to moderate heat and cold stress. We demonstrate that the REEAD sensor system enables measurement of a reduced TOP1 activity in crude extracts from *D. melanogaster* in response to both short-term and long-term heat stress. The obtained results demonstrate that TOP1 activity may be a new marker for heat stress response in flies and suggest a new utilization of the REEAD sensor system in addressing heat stress responses at the organism level. In human cells, we observed reduced TOP1 activity levels in response to both heat and cold stress. Similar effects were not observed upon subjecting purified TOP1 to similar conditions before measuring activity, indicating that the reduced TOP1 activity relies on cellular stress responses. Finally, by adapting the REEAD sensor system to a quantitative chemiluminescence readout we point towards the development of a sensor system suitable for point-of-care assessment of stress responses based on measurement of TOP1 activity in crude biological samples.

## 2. Materials and Methods

### 2.1. Reagents

All chemicals, cell media, and media components were purchased from Merch Life Sciences A/S (Søborg, Denmark). TRIDIA HD slides (DHD1-0023) were from SurModics (Saint Paul, MN, USA), Vectashield without DAPI (H-1000) was from Vector Laboratories (Burlington, ON, Canada), and the synthetic gene of the Phi29 polymerase was from GenScript (Piscataway, NJ, USA). Ceramic SiLiBeads were from Sigmund Lindner (Warmensteinach, Germany).

### 2.2. DNA Substrates and Oligonucleotides

DNA oligonucleotides were synthesized by Merch Life Sciences A/S (Søborg, Denmark). The sequences of the oligonucleotides were as follows:

RCA primer: 5′-[AmC6] CCA ACC AAC CAA CCA AAT AAG CGA TCT TCA CAG T-3′

TOP1 substrate: 5′-AGA AAA ATT TTT AAA AAA ACT GTG AAG ATC GCT TAT TTT TTT AAA AAT TTT TCT AAG TCT TTT AGA TCC CTC AAT GCA CAT GTT TGG CTC CGA TCT AAA AGA CTT AGA-3′

Detection oligo: 5′-[6FAM] CCT CAA TGC ACA TGT TTG GCT CC-3′

TOP1 qPCR forward primer: 5′-ACTACTTCCAAGCGGAGTCC

TOP1 qPCR reverse primer: 5′-AAGTTACCGATCTTCTCCTTG

Actin qPCR forward primer: 5′-GCG TCG GTC AAT TCA ATC TT

Actin qPCR reverse primer: 5′-AAG CTG CAA CCT CTT CGT CA

### 2.3. Fly Stock and Maintenance

*D. melanogaster* were from Bloomington Drosophila Stock Center (Indiana University, Bloomington, IN, USA) (ID number and genotype: 28240 (ISO40) (genotype DGRP-812/RAL-812), 55014 (ISO14) (genotype DRGP-31/RAL-31), 28213 (ISO13) (genotype DRGP-589/RAL-589)) [55]. The flies were subjected to several generations of full sibling inbreeding of the progeny. Odder2013 flies used for this study as wildtype flies originated from an apple orchard 30 km south of Aarhus (55°56′42.46″ N, 10°12′45.31″ E), where 25 female flies were caught in 2013 and since kept at intermediate densities in the laboratory. All experiments were conducted on standard oatmeal-sugar-yeast-agar Drosophila medium.

Flies were reared at 25 °C and kept at 12/12 h light/dark cycle at low densities. The experiments were performed on male flies collected after 10 days post fertilization by means of CO_2_ anesthesia. The male flies were kept on standard medium with a density of 12 males in 15 mL Falcon centrifuge tubes (note, we stressed 12 flies and used 8 flies for activity measurements. The reason for stressing more than needed was that in some cases a few flies were lost during the stress process for different reasons unrelated to the stress). The Falcon tubes containing the flies were capped with a water-repellent cotton wool lid except during heat stress treatment.

### 2.4. Thermal Stress of D. melanogaster

To examine the effect of heat stress on male flies, the experimental groups were subjected to 2 h (short-term) or 6 days (long-term) of heat stress in water baths set at different temperatures. The experimental temperatures were 31 °C, 33 °C, 35 °C, and 37 °C. The control group was subjected to 25 °C water bath. During treatment in water baths the cotton wool lid of the tubes was replaced with the original screw cap to avoid water from entering the Falcon tubes. After heat stress, the experimental groups and control groups were moved to a 25 °C temperature-regulated room for stress recovery of 1, 4, 16, 48, or 96 h. The flies were sacrificed and frozen at −80 °C in Eppendorf tubes after stress recovery.

### 2.5. Cell Culture

The human cell line HeLa (subtype CCL2 from ATCC, Manassas, VA, USA [56]) was cultured in Dulbecco’s Modified Eagle Medium (DMEM, Gibco, New York, NY, USA) supplemented with 10% fetal bovine serum (FBS, Sigma-Aldrich, St. Louis, MO, USA), and 100 units/mL penicillin/100 mg/mL streptomycin (Life Technologies, Carlsbad, CA, USA). The cells were maintained in a humidified incubator (5% CO_2_/95% air atmosphere at 37 °C).

### 2.6. Thermal Stress of HeLa Cells

HeLa cells were seeded in culture flasks and cultured at 37 °C one day prior to thermal stress treatments. At 70–80% confluency, the cells were moved to incubators preheated or precooled to 37 °C (control group), 40 °C, 42 °C, 22 °C or 4 °C for a duration of 3 h. Following thermal stress, the cells were moved to 37 °C for 1 h of recovery. The cells were harvested by trypsination (0.25% Trypsin-EDTA solution) and stored at −80 °C until further analysis.

### 2.7. Preparation of Cell Extract for Molecular Analyses

#### 2.7.1. HeLa

Cell pellets were obtained by harvest as described above (see “Thermal stress of HeLa cells”). The pellets were lysed in 10 mM Tris-HCl pH 7.5, 5 mM EDTA for 10 min on ice. Following incubation, the extract was thoroughly mixed by pipetting and diluted appropriately for subsequent analyses.

#### 2.7.2. *D. melanogaster*

8 male flies were subjected to tubes containing 10 mM Tris-HCl pH 7.5, 5 mM EDTA supplemented with protease inhibitor and 1.4–1.6 mm ceramic beads. Extraction was performed by using a bead-beating instrument (Precellys, Bertin Instruments, Fontaine, France) for 2× 20 s at 6300 rpm, 4 °C. The supernatant was obtained after centrifugation of the solution at 960× *g* for 30 s, 4 °C, and used directly in subsequent analyses.

### 2.8. REEAD Assay

#### 2.8.1. Preparation of the Slides

TRIDIA HD activated slides were coupled with 25 pmol RCA primer specific for the TOP1 substrate in print buffer (300 mM Na_3_PO_4_ pH 8) and incubated overnight in a humidity chamber with saturated NaCl at room temperature. The slides were blocked for 30 min in 50 mM Tris, 50 mM Tris-HCl pH 9, 50 mM ethanolamine at 50 °C, and subsequently washed for 30 min in 4×SSC, 0.1% SDS at 50 °C.

#### 2.8.2. Circularization, Rolling Circle Amplification, and Detection

The circularization reaction was carried out in a total volume of 20 µL in 1×TE+E (10 mM Tris-HCl pH 7.5, 5 mM EDTA) supplemented with 1 pmol (HeLa cells and purified hTOP1) or 200 pmol (*D. melanogaster*) of TOP1 specific substrate and 50 mM NaCl. The reaction was initiated by addition of 2 µL cell extract of either control samples or stress treated samples (see “Preparation of cell extract for molecular analyses”). The samples were incubated at 37 °C (HeLa cells and purified hTOP1) or 25 °C (*D. melanogaster*) for 30 min. The closed circles were hybridized to the surface anchored RCA primer for 1 h at 37 °C. Following hybridization, the slides were washed in wash buffer 2 (100 mM Tris-HCl pH 7.5, 150 mM NaCl, 0.3% SDS) for 1 min at room temperature, wash buffer 3 (100 mM Tris-HCl pH 7.5, 150 mM NaCl, 0.05% Tween-20) for 1 min at room temperature and finally dehydrated in 70% EtOH for 1 min at room temperature. Subsequently, rolling circle amplification synthesis was carried out for 1 h at 37 °C. The reaction was performed in 1× Phi29 reaction buffer (50 mM Tris-HCl, pH 7.5, 10 mM MgCl_2_, 10 mM (NH_4_)_2_SO_4_, 4 mM DTT) supplemented with 10 µg/µL BSA, 10 mM dNTP, and 10 units/µL Phi29 polymerase. The reaction was stopped by washing the slide in wash buffer 2 and 3 for 1 min at room temperature followed by dehydration in 70% EtOH for 1 min at room temperature. Detection of the rolling circle products was obtained by hybridization of 0.2 µM of the detection probe in a 1× hybridization buffer (20% formamide, 2×SSC, 5% glycerol) for 30 min at 37 °C. The reaction was again stopped by washing the slide in wash buffer 2 for 15 min and 3 for 5 min at room temperature followed by dehydration in 70% EtOH for 1 min at room temperature. The slides were finally mounted with Vectashield and visualized using a 60× objective in an Olympus IX73 fluorescence microscope. The signals detected in an average of 10 microscopic images were counted using Fiji ImageJ and analyzed in GraphPad Prism software v10.

### 2.9. Heat Denaturation of Purified Human Topoisomerase 1

Purified hTOP1 was incubated at either 37 °C, 42 °C, 65 °C, or 4 °C for 2 h following 1 h at 37 °C for recovery. From each condition, 0.16 ng of hTOP1 was added to a reaction mixture containing 1× TE+E (10 mM Tris-HCl pH 7.5, 5 mM EDTA), 50 mM NaCl, and 1 pmol TOP1 specific substrate. The circularization of the substrate was performed at 37 °C for 30 min, and the samples were stored at −20 °C before further analysis. The remaining of the REEAD assay was performed as described above (see Section 2.8).

### 2.10. Western Blot

Whole cell extracts from HeLa cells were mixed with SDS loading buffer (2% SDS, 2 mM β-mercaptoethanol, 5% (*v*/*v*) glycerol, 50 mM Tris-HCl pH 7, 0.05% bromophenol blue) and loaded onto a 10–20% Novex Tris-Glycine mini gel. Electrophoresis was performed at 200 V for 45 min in 1× SDS running buffer (25 mM Tris-HCl, 192 mM glycine, 0.1% SDS) and subsequently blotted onto a 0.2 µm nitrocellulose membrane (Amersham Protran, GE Healthcare, Brøndby, Denmark) at 100 V for 1.5 h at 4 °C in 25 mM Tris-HCl, 0.1% SDD, 10% ethanol. The membrane was blocked for 1 h at room temperature in 20 mM Tris-HCl, 0.5 M NaCl, 0.0225% Tween20 supplemented with 5% skimmed milk powder. Subsequently, incubation with primary antibody was performed overnight at 4 °C using a TOP1 polyclonal antibody (1:3000, Bethyl Laboratories, Montgomery, TX, USA) and TATA-binding protein (TBP, 1:2000, Abcam, Cambridge, UK). The membrane was washed 3× 30 min in 1×TBST (20 mM Tris pH 7.5, 0.5 M NaCl, 0.25% Tween20) at room temperature and probed for 1 h at room temperature with secondary antibodies (HRP conjugated goat anti-rabbit (1:2000) or goat anti-mouse (1:1500), Dako, Glostrup, Denmark). The membrane was washed 3× 15 min in 1×TBST at room temperature. Labelled antibodies were detected by autoradiography using ECL Plus Western Blotting Detection Reagents (GE Healthcare) before exposure to Amersham Hyperfilm ECL (GE Healthcare, Chicago, IL, USA).

### 2.11. Quantitative PCR

RNA from *D. melanogaster* was purified using RNeasy mini kit (Qiagen, Hilden, Germany) following the manufacturer’s instructions. The purified RNA was used for cDNA synthesis using QuantiTect Reverse Transcription kit (Qiagen) following the manufacturer’s instruction. All qPCR reactions were performed using Platinum SYBR Green qPCR SuperMix (Invitrogen, ThermoFisher Scientific, New York, NY, USA) and actin was used as a reference gene. For each specific primer pair, a qPCR master mix containing 7.5 µL SYBR Green, 0.03 µL ROX reference dye, 1 µL 2 µM forward and reverse primers, and 0.47 µL H_2_O was prepared. Each reaction was performed in triplicate with 5 ng cDNA. The qPCR reaction was performed on a Stratagene M×3000P (Agilent Technologies, Santa Clara, CA, USA) using the following program: 15 min at 95 °C, 40 cycles of 30 s at 95 °C, 1 min at 56 °C, 40 s at 72 °C. The run was ended with 1 min at 95 °C, 30 s at 56 °C, and 30 s at 95 °C. The amount of TOP1 was calculated based on the Ct values using the following equation: ΔCt = Ct(target) − Ct(reference) and ΔCtexp = 2^−ΔCt^.

### 2.12. Protein Purifications

#### 2.12.1. Human Topoisomerase 1

The RS190 yeast *S*. *cerevisiae* strain was transformed with the expression plasmid pHT143, for expression and purification of hTOP1. The protein was expressed, and either crude extracts or purified enzyme was prepared as previously described [57,58]. The eluted protein was analyzed on a 12% SDS PAGE followed by visualization with Coomassie Brilliant Blue staining.

#### 2.12.2. Phi29 Polymerase

The synthetic gene for Phi29 polymerase (GenScript, Singapore) was cloned into the pGEX vector generating in a recombinant N-terminal GST-tagged Phi29 polymerase expression plasmid.

*E. coli* competent BL21 cells (Promega, Singapore) were transformed with the plasmid for expression and purification of Phi29 polymerase. Cultivation was carried out in 2×TY media supplemented with 100 µg/mL ampicillin and expression was induced in log phase at OD600 = 0.8 using 1 mM IPTG at 37 °C for 2 h. The cells were then harvested, followed by resuspension in sonication buffer (50 mM Tris-HCl pH 7.5, 2.5 M NaCl, 1 mM EDTA, 1 mM DTT, 10 mg/mL Lysozyme). After 1 h of incubation on ice, the cells were lysed by freeze and thawing in liquid N_2_ followed by sonication. The lysate was then mixed with 4% Steptomycin Sulfate and incubated for 1 h at 4 °C. Using centrifugation, insoluble particles were removed from the lysate before filtration through a 0.45 µm filter and subsequently loaded onto a pre-equilibrated GST Gravitrap column (GE Healthcare) following manufacturer’s instructions. The column was washed in 10× columns of sonication buffer and protein eluted in 10× column volumes of elution buffer (10 mM Tris-HCl pH 8.5, 5 mM Glutathione, 500 mM NaCl). The eluted protein was collected in fractions and analyzed on a 12% SDS PAGE and visualized by Coomassie Brilliant Blue staining. Finally, the protein fractions were dialyzed against 50% glycerol, 0.5% Tween20, 1 mM DTT, and 0.5% NP40 at 4° overnight.

### 2.13. Statistical Analyses

Statistical analysis of two group means was conducted using unpaired, two-tailed students *t*-test and the analysis of the mean from three or more groups was obtained using unpaired one-way ANOVA test applying Brown-Forsythe and Welch correction. Data were analyzed using GraphPad Prism 10 software (La Jolla, San Diego, CA, USA) and expressed as mean +/− standard error of the mean (SEM).

## 3. Results and Discussion

### 3.1. Short-Term Heat Stress Leads to Reduced TOP1 Activity Levels in D. melanogaster

**Figure 1:**
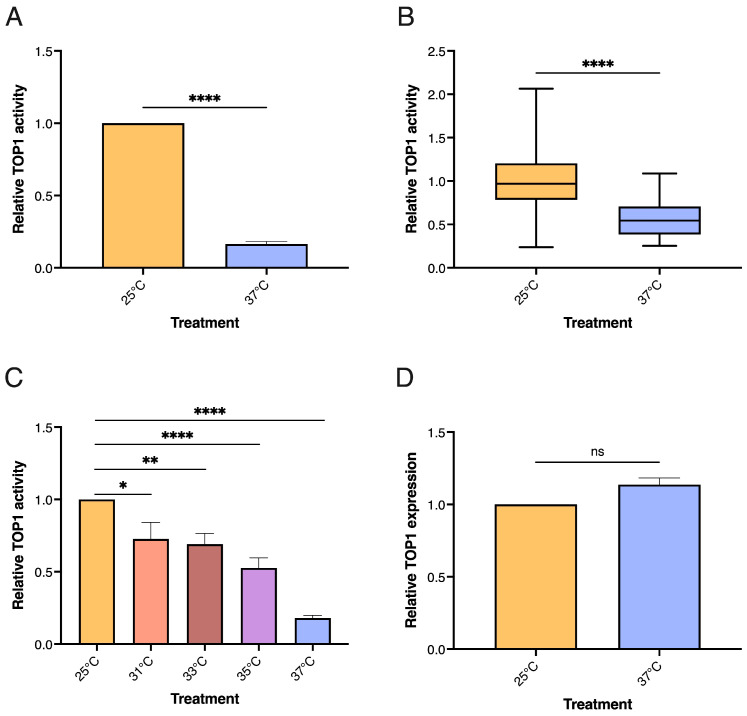
TOP1 activity in *D. melanogaster* after exposure to acute/short-term heat stress. (**A**) Graphical depiction of TOP1 activity measured by REEAD in whole extract from a group of 8 male flies after exposure to acute/short-term stress at 37 °C for 2 h followed by 1 h of recovery at 25 °C. A control group kept at 25 °C is included, n = 14. (**B**) Boxplot showing the TOP1 activity measured by REEAD in extracts from 46 single male flies after exposure to acute/short-term heat stress or 25 °C as in (**A**). (**C**) Graphical depiction of TOP1 activity measured by REEAD in extracts from a group of 8 male flies exposed to increasing temperatures (25 °C, 31 °C, 33 °C, 35 °C, or 37 °C) for 2 h followed by 1 h recovery at 25 °C, n = 14. (**D**) mRNA levels of TOP1 measured by qPCR in extracts from a group of 8 male flies exposed to acute/short-term stress or 25 °C as in (**A**), n = 3. Data in all graphical depictions are normalized to the unstressed control group at 25 °C and plotted at mean +/− standard error of the mean (SEM). * Indicates significant differences, * *p* < 0.05; ** *p* < 0.005; **** *p* < 0.0001; ns = non-significant.

To investigate the effects of short-term heat stress in *D. melanogaster* in terms of TOP1 activity levels, populations of male flies were heat stressed at 37 °C for 2 h and sacrificed after 1 h recovery at 25 °C. For each population subjected to heat stress a control group kept at 25 °C was included. To avoid different background responses due to genetic variations we used three isogenic lines (ISO13, ISO14, and ISO40) generated by inbreeding [55]. Groups of 8 flies from each population of stressed or unstressed flies were sedated and sacrificed by flash freezing in dry ice. The cellular content from the 8 flies combined, was extracted by bead beating as described in materials and methods. TOP1 activity in whole extracts from the different groups of flies was measured using the REEAD sensor system, that allows sensitive, accurate, and quantitative measurement of individual TOP1 cleavage/ligation events in crude samples [54]. The results combined from all three *D. melanogaster* stains are graphically depicted in Figure 1A and demonstrate a significant and pronounced reduction in TOP1 activity after exposure to heat stress at 37 °C for 2 h.

When comparing the response between flies from the three different lines (Supplementary Figure S1) the response appears very similar regardless of the genetic background suggesting that the reduced TOP1 activity levels are independent of genetics. Also, when comparing the response in single flies it is evident that stressed flies are characterized by a reduced TOP1 activity level compared to unstressed flies. As indicated in the boxplot shown in Figure 1B, the median of the TOP1 activity levels measured in extracts from the stressed individuals differed significantly from the median of the TOP1 activity levels measured in extracts from unstressed individuals. These results suggest the potential applicability of TOP1 activity levels as a marker for stress in individual flies.

Less pronounced, yet significant, reduction in TOP1 activity was observed when the flies were subjected to less severe heat stress conditions (31–35 °C), as evident from the results combined from the three lines shown in Figure 1C. The reduced TOP1 activity levels were not accompanied by a reduced expression of TOP1 as heat stress treatment did not affect the TOP1 mRNA levels measured by qPCR (see Figure 1D). Hence, the stress-induced alterations of TOP1 activity are more likely caused by posttranslational modifications [59,60,61], intracellular metabolites that may affect enzyme activity [62] or protein-protein interactions [63,64,65], which are well known to modulate TOP1 activity.

**Figure 2:**
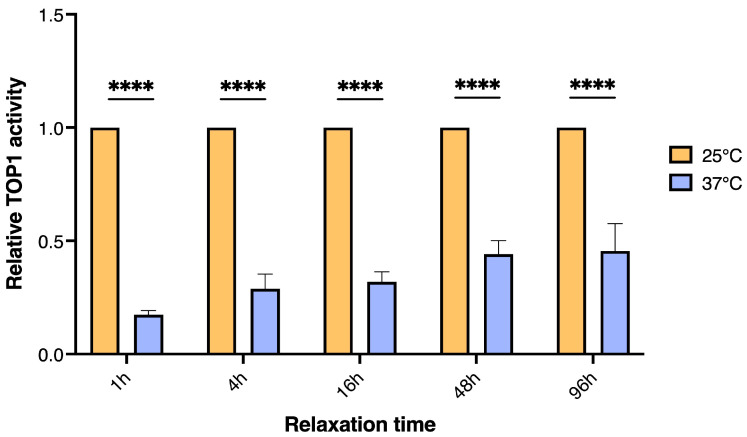
TOP1 activity in *D. melanogaster* after acute/short-term heat stress with prolonged recovery times. Graphical depiction of TOP1 activity measured by REEAD in extract from 8 *D. melanogaster* exposed to acute/short-term heat stress at 37 °C for 2 h followed by increasing recovery times from 1 to 96 h (blue bars). Data are normalized to the TOP1 activity measured in extracts from the unstressed control group (orange bars) kept at 25 °C for the duration of the experiment and plotted at mean +/− SEM, n = 10. * Indicates significant differences, **** *p* < 0.0001.

In cultivated human cells, Velichko et al. (2015) have previously demonstrated full recovery in terms of TOP1cc removal after 6 h at 3 °C following severe short-term heat stress (45.5 °C for 30 min) [49]. We wanted to investigate if the TOP1 activity levels were restored after prolonged recovery times after short-term heat stress in *D. melanogaster*. Populations of male flies were treated at 37 °C for 2 h and placed at 25 °C for recovery for 1, 4, 16, 48, and 96 h, respectively, before they were sedated and sacrificed by flash freezing as described above. Groups of 8 flies from each population were bead beaded and the TOP1 activity level in whole fly extracts was measured by REEAD. As evident from the graphical depiction of the results (Figure 2), the TOP1 activity level decreased dramatically after heat stress followed by 1 h recovery at 25 °C which is consistent with the results shown in Figure 1A. Even though the activity level of TOP1 did increase with increasing recovery intervals, only approx. 45% of the basal level (the activity level measured in unstressed control flies kept at 25 °C) was reached after 96 h of recovery. This demonstrated a rather persistent reduction in TOP1 activity levels after stress treatment.

### 3.2. The Effect of Long-Tern Heat Stress in D. melanogaster on TOP1 Activity

Evidence from stress studies in various organisms indicate that the cellular and physiological responses may differ between short- and long-term stress [66]. In physiology, short-term exposure to high temperatures is often referred to as heat hardening, while short-term exposure to cold temperatures is referred to as cold hardening [67]. Both types of hardening usually increase tolerance to even more extreme temperatures afterward [68]. For instance, studies demonstrate that even though high cortisol levels are a well-known marker for acute (short-term) stress, cortisol levels may stabilize at basal levels as a response to chronic stress (long-term) in *Danio rerio* [69]. The lack of a central nervous system renders the definitions of acute or chronic stress in *D. melanogaster* irrelevant. However, short- and long-term stress may pose different challenges even in fruit flies and we therefore wanted to expand our studies to encompass flies exposed to prolonged heat stress. However, if the stress is high, organisms/flies may only survive a certain level of total stress, which is the cumulative stress experienced over a period.

**Figure 3:**
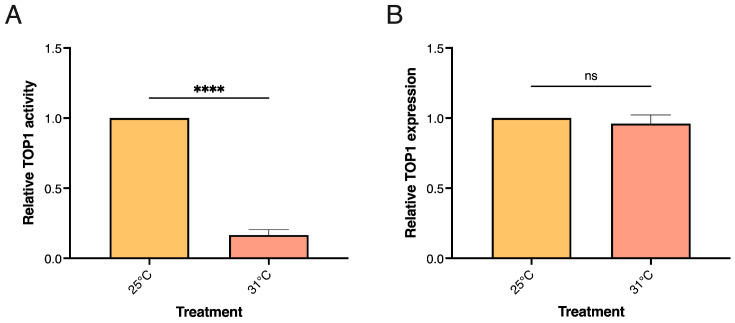
TOP1 activity and expression in *D. melanogaster* exposed to long-term heat stress. (**A**) Graphical depiction of TOP1 activity measured by REEAD in extract from a group of 8 male flies exposed to long-term heat stress at 31 °C for six days followed by 1 h of recovery at 25 °C. A control group kept at 25 °C is included, n = 12. (**B**) mRNA levels of TOP1 measured by qPCR in extracts from a group of 8 male flies exposed to long-term heat stress or 25 °C as in (**A**), n = 3. Data are normalized to the unstressed control group kept at 25 °C and plotted at mean +/− SEM. * Indicates significant differences, **** *p* < 0.0001; ns = non-significant.

In *D. melanogaster* long-term heat stress conditions are less investigated than short-term stress conditions. Therefore, the best possible temperature exposures were established by determining the highest possible temperature that allowed survival of >90% of the flies after 6 days of exposure. The flies were exposed to temperatures ranging from 31 to 37 °C with 2 °C intervals. Survival of >90% of the flies was observed after treatment at 31 °C but not after exposure to higher temperatures. Therefore, the experiment was performed under this condition. Wildtype (Odder2013) or isogenic inbred lines (ISO14 or ISO40) of male flies were treated at 25 °C (as a control) or 31 °C for 6 days before they were sedated, divided into groups of 8, flash frozen and subjected to bead beading to extract active enzyme. TOP1 activity in whole fly extracts from the different groups was measured by REEAD and the results combined for all populations shown in Figure 3A.

As evident from the graphical depiction, the TOP1 activity level was dramatically decreased after exposure to long-term heat stress as it was the case after short-term heat stress. Also, after long-term heat stress the decreased activity level of TOP1 could not be ascribed to decreased expression since the TOP1 mRNA levels were comparable in stressed versus unstressed flies when measured by qPCR (Figure 3B). Combined all three lines investigated responded to prolonged heat stress by a significant reduction in TOP1 activity, however, when comparing the response between the individual lines (Supplementary Figure S2) it is evident that the isogenic inbred lines (ISO14 and ISO40) appeared more sensitive to heat stress and showed a more pronounced downregulation of TOP1 activity upon heat stress than the wildtype line. Wildtype/outbreed flies are in general less sensitive to stress than inbred flies, which is also evident by the induction of heat-stress marker hsp70 at lower temperatures in inbred compared to wildtype flies. Inbreeding itself is already stressful [70].

### 3.3. TOP1 Activity Levels in Human Cells Are Reduced in Response to Short-Term Cold and Heat Stress

**Figure 4:**
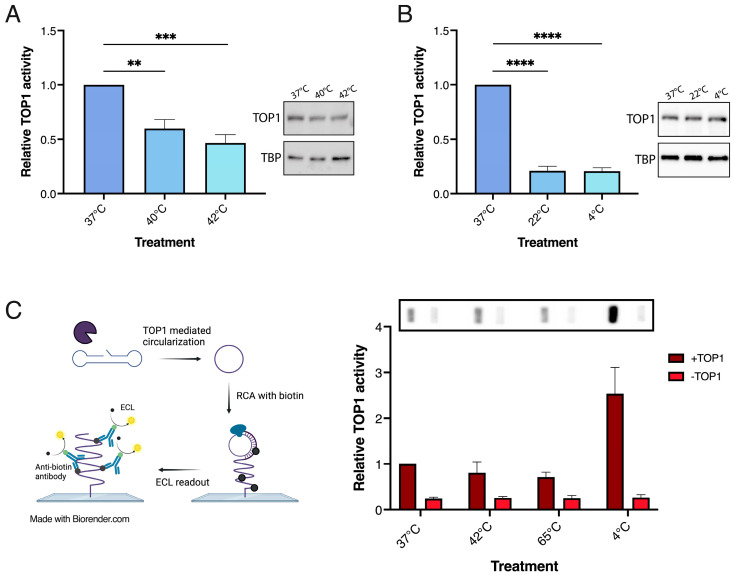
TOP1 activity in HeLa cells after exposure to acute heat or cold stress. (**A**) Left panel: Graphical depiction TOP1 activity measured by REEAD in whole cell extract from HeLa cells exposed to optimal temperature (37 °C) or heat stress at 40 °C or 42 °C for 2 h followed by 1 h of recovery at 37 °C, n = 7. Right panel: Western blot indicating TOP1 expression levels after exposure to 37 °C or heat stress at 40 °C or 42 °C. Top bands correspond to TOP1, lower bands correspond to TBP loading control. (**B**) Same as (**A**), except the cells were exposed to cold stress at 22 °C or 4 °C, n = 7. Data in (**A**,**B**) are normalized to the unstressed group at 37 °C and plotted at mean +/− SEM. * Indicates significant differences, ** *p* < 0.005; *** *p* < 0.0005; **** *p* < 0.0001. (**C**) Left panel: Schematic illustration of the REEAD assay using an ECL based readout. The TOP1 specific substrate is cleaved and ligated by TOP1 before amplification by RCA in the presence of biotin-coupled nucleotides. HRP conjugated anti-biotin antibodies are bound to the biotinylated products allowing for visualization using ECL as a substrate for HRP, resulting in a chemiluminescent product. Right top panel, representative image obtained after analyzing purified hTOP1 activity after exposure to increasing temperatures from 4 °C to 65 °C for 2 h followed by 1 h recovery at 37 °C by REEAD using the ECL based readout. Right bottom panel, quantification of the results shown at the top panel. Data are normalized to the control group kept at 37 °C, n = 4.

As mentioned, previous studies demonstrated a reduced TOP1 activity level in cultivated human HeLa cells upon exposure to heat stress. To investigate if a similar response could be observed by using the REEAD sensor system for measuring TOP1 cleavage/ligation activity, HeLa cells were cultivated to 70–80% confluency before they were exposed to heat stress at either 40 or 42 °C (or to 37 °C as a control) for 2 h followed by 1 h recovery time at 37 °C. The cells were pelleted and lysed by the addition of a hypotonic buffer to generate whole cell extracts as previously described [71]. As evident in Figure 4A treatment at moderately increased temperatures to either 40 or 42 °C significantly reduced the activity levels of TOP1 (left panel), without affecting the expression levels (right panel), which is in line with previous reports.

Increased temperatures may lead to decreased activity or functionality of enzymes or proteins simply by hampering the correct folding of the proteins. To investigate if the observed reduction of TOP1 activity was unique to heat stress, we investigated the influence of cold stress on TOP1 activity in HeLa cells. As for the investigations of heat stress cells were cultivated to 70–80% confluency and exposed to cold stress at either 22 °C or 4 °C for 2 h following 1 h recovery at 37 °C. A control group that was kept at 37 °C for the duration of the treatment was included. The TOP1 activity in whole cell extracts was measured as described above, and the results are graphically depicted in Figure 4B. A pronounced reduction of TOP1 activity (left panel) but not expression (right panel) was observed both after exposure to 22 and 4℃, indicating that the effect on TOP1 was not unique to heat stress. This observation argues against the reduced TOP1 activity being the result of protein denaturation. This was confirmed by investigating the effect of subjecting purified hTOP1 to various temperatures for 2 h followed by a recovery period of 1 h at 37 °C before activity was measured. As evident from Figure 4C, preincubation of purified TOP1 at either 42 or 65 °C did not change the activity significantly when compared to the activity of purified TOP1 preincubated at 37 °C. Preincubation at 4 °C, on the other hand, resulted in a markedly increased activity compared to the enzyme preincubated at 37 °C. This most probably reflects a better preservation of the enzyme activity when kept at 4 °C rather than higher temperatures. Hence, we conclude that the reduced TOP1 activity in response to thermal stress in human cells is a consequence of one or more cellular pathways affecting TOP1 activity rather than a direct physical effect (e.g., denaturation) of the enzyme upon exposure to increased or reduced temperature. These observations taken together with the possibility of quantitatively measuring TOP1 activity using the gel-free and simple-to-use REEAD sensor system combined with an easily accessible chemiluminescence readout (see [72] for further method description) as exemplified in Figure 4C point towards a possibility of using of TOP1 as a biomarker for stress.

As mentioned, a variety of stressors induce similar responses at the cellular level arguing for the possibility of identifying suitable markers for monitoring stress in general and thermal stress imposed by changing temperatures in particular [73]. However, until now most biological markers have been identified and validated in studies of acute or severe stress. Little is known about the cellular responses toward long-term low to moderate stress that can be part of everyday life.

A few previous studies have implicated nuclear processes and enzymes such as TOP1 in the cellular heat stress response using cultivated human cells as a model system [12,74]. In the present study, we demonstrated a markedly reduced TOP1 activity level in cultivated human cells in response to both severe and moderate heat stress. This response was not a consequence of simple heat denaturation of the enzyme, since a similar activity decrease was not observed when the purified enzyme was subjected to similar temperature increases in a cell-free system and since cold treatment of the cells also resulted in a decreased TOP1 activity level. The TOP1 protein level was not affected by the treatment, suggesting that thermal stress in human cells affects the activity but not the expression or stability of the enzyme. Although human cells may be used as a model for various cellular processes it may be a less optimal system for studying stress, as physiological factors may be pivotal for stress responses in multicellular organisms [75,76]. We, therefore, addressed the TOP1 activity levels in the multicellular organism *D. melanogaster* in response to short-term severe as well as long-term mild to moderate heat stress and observed in all cases a significantly reduced TOP1 activity level in response to the stress treatment. This response was rather stable and although some level of recovery could be observed, a significantly reduced TOP1 activity level persisted even 96 h post-treatment.

## 4. Conclusions

The data presented in the current manuscript suggest the potential use of TOP1 activity as a stable biomarker for both short-term severe and long-term moderate stress in the simple organism *D. melanogaster* and in human HeLa cells. When measuring the TOP1 activity at the single fly level, we demonstrated a significant difference between populations of stressed versus unstressed flies. This result supports the potential use of TOP1 activity as a novel stress marker. We also demonstrate that TOP1 activity in crude extracts from stressed or unstressed flies or human cells can be measured by the easily accessible and quantitative REEAD sensor system further adding to the potential values of TOP1 activity as a future marker for thermal stress.

## Data Availability

Data sharing is not applicable to this article.

## Supplementary Materials

The following supporting information can be downloaded at: https://www.mdpi.com/article/10.3390/bios13110950/s1, Figure S1: TOP1 activity in *D. melanogaster* strains after exposure to acute/short-term heat stress; Figure S2: TOP1 activity in *D. melanogaster* strains after exposure to long-term heat stress.
