# Supplementary material for: Topoisomerase 1 Activity Is Reduced in Response to Thermal Stress in Fruit Flies and in Human HeLa Cells
Source: Biosensors (Basel). 2023 Oct 24;13(11):950. doi: 10.3390/bios13110950 (PMC10669382; doi:10.3390/bios13110950)
Supplement: Supplementary file 1 [file biosensors-13-00950-s001.zip › biosensors-2652759-supplementary.pdf]

biosensors

MDPI

Supplementary Materials

Topoisomerase 1 Activity Is Reduced in Response to Thermal Stress in Fruit Flies and in Human HeLa Cells

Trine Juul-Kristensen [1,†], Josephine Geertsen Keller [1,†], Kathrine Nygaard Borg [1,2], Noriko Y. Hansen [1], Amalie Foldager [1], Rasmus Ladegaard [1], Yi-Ping Ho [2,3,4,5], Volker Loeschcke [6,*] and Birgitta R. Knudsen [1,*]

[1] Department of Molecular Biology and Genetics, Aarhus University, 8000 Aarhus, Denmark; tjk@mbg.au.dk (T.J.-K.); jgk@mbg.au.dk (J.G.K.); kborg@link.cuhk.edu.hk (K.N.B.); noriko@mbg.au.dk (N.Y.H.); afo@clin.au.dk (A.F.); ladegaardmadsen@gmail.com (R.L.)
[2] Department of Biomedical Engineering, The Chinese University of Hong Kong, Hong Kong SAR, China; ypho@cuhk.edu.hk
[3] Centre for Biomaterials, The Chinese University of Hong Kong, Hong Kong SAR, China
[4] Hong Kong Branch of CAS Center for Excellence in Animal Evolution and Genetics, Hong Kong SAR, China
[5] State Key Laboratory of Marine Pollution, City University of Hong Kong, Hong Kong SAR, China
[6] Department of Biology, Aarhus University, 8000 Aarhus, Denmark
* Correspondence: volker@bio.au.dk (V.L.); brk@mbg.au.dk (B.R.K.)
† These authors contributed equally to the presented work.

1. Supplementary Figures

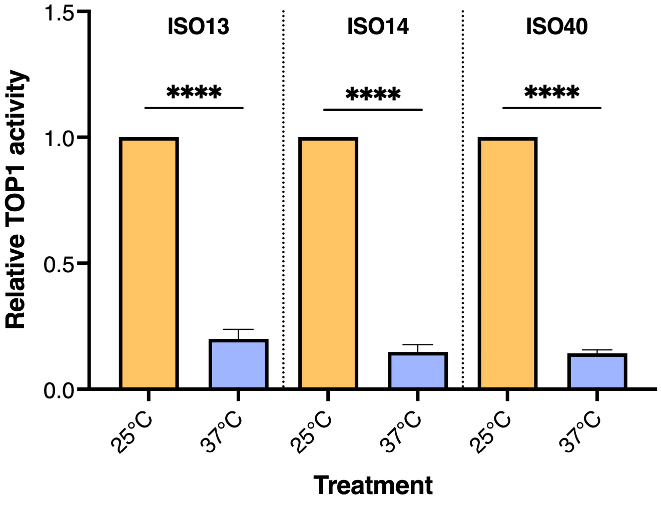

Figure S1. TOP1 activity in *D. melanogaster* strains after exposure to acute/short-term heat stress. Graphical depiction of TOP1 activity measured by REEAD in extract from a group of eight male flies from either ISO13, ISO14, or ISO40 strain. The flies were exposed 25°C or to acute/short-term heat stress at 37°C for 2 hours followed by 1 hour of recovery at 25°C, n=5. Data are normalized to the unstressed group at 25°C for each separate strain and plotted at mean +/- SEM. * Indicates significant differences, **** $p<0.0001$.

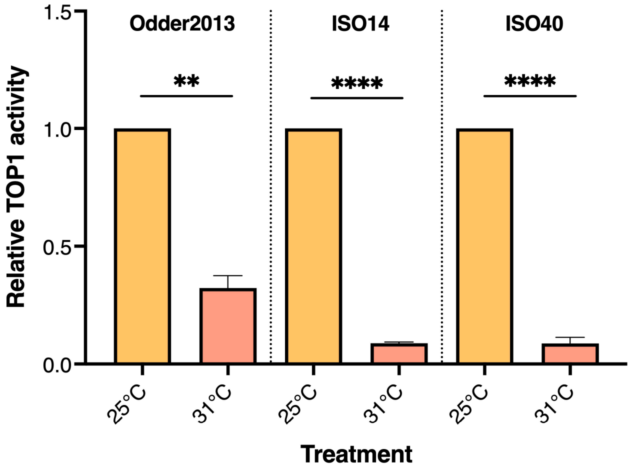

Figure S2. TOP1 activity in *D. melanogaster* strains after exposure to long-term heat stress. Graphical depiction of TOP1 activity measured by REEAD in extract from a group of eight male flies from either Odder2013, ISO14, or ISO40 strain. The flies were exposed to 25°C or long-term heat stress at 31°C for six days followed by 1 hour of recovery at 25°C, n=4. Data are normalized to the unstressed group at 25°C for each separate strain and plotted at mean +/- SEM. * Indicates significant differences, ** $p<0.005$; **** $p<0.0001$.
